# Extensive Variation in Gene Expression is Revealed in 13 Fertility-Related Genes Using RNA-Seq, ISO-Seq, and CAGE-Seq From Brahman Cattle

**DOI:** 10.3389/fgene.2022.784663

**Published:** 2022-03-25

**Authors:** Elizabeth M. Ross, Hari Sanjana, Loan T. Nguyen, YuanYuan Cheng, Stephen S. Moore, Ben J. Hayes

**Affiliations:** ^1^ Centre for Animal Science, Queensland Alliance for Agriculture and Food Innovation, The University of Queensland, St Lucia, QLD, Australia; ^2^ School of Life and Environmental Sciences, The University of Sydney, Sydney, NSW, Australia

**Keywords:** fertility, cattle, gene expression, transcriptomics, variation, isoforms, androgen receptor, RNA sequencing

## Abstract

Fertility is a key driver of economic profitability in cattle production. A number of studies have identified genes associated with fertility using genome wide association studies and differential gene expression analysis; however, the genes themselves are poorly characterized in cattle. Here, we selected 13 genes from the literature which have previously been shown to have strong evidence for an association with fertility in Brahman cattle (*Bos taurus indicus*) or closely related breeds. We examine the expression variation of the 13 genes that are associated with cattle fertility using RNA-seq, CAGE-seq, and ISO-seq data from 11 different tissue samples from an adult Brahman cow and a Brahman fetus. Tissues examined include blood, liver, lung, kidney, muscle, spleen, ovary, and uterus from the cow and liver and lung from the fetus. The analysis revealed several novel isoforms, including seven from *SERPINA7*. The use of three expression characterization methodologies (5′ cap selected ISO-seq, CAGE-seq, and RNA-seq) allowed the identification of isoforms that varied in their length of 5′ and 3′ untranslated regions, variation otherwise undetectable (collapsed as degraded RNA) in generic isoform identification pipelines. The combinations of different sequencing technologies allowed us to overcome the limitations of relatively low sequence depth in the ISO-seq data. The lower sequence depth of the ISO-seq data was also reflected in the lack of observed expression of some genes that were observed in the CAGE-seq and RNA-seq data from the same tissue. We identified allele specific expression that was tissue-specific in *AR*, *IGF1*, *SOX9*, *STAT3*, and *TAF9B*. Finally, we characterized an exon of *TAF9B* as partially nested within the neighboring gene phosphoglycerate kinase 1. As this study only examined two animals, even more transcriptional variation may be present in a genetically diverse population. This analysis reveals the large amount of transcriptional variation within mammalian fertility genes and illuminates the fact that the transcriptional landscape cannot be fully characterized using a single technology alone.

## Introduction

In tropical regions, *Bos taurus indicus* and crosses between *Bos taurus indicus* and *Bos taurus taurus* are extensively used as they are more resistant to heat stress, diseases, and ticks. Brahman cattle are a *Bos taurus indicus* breed extensively raised in tropical regions, including Northern Australia, Brazil, South Asia, and North America. In tropical beef production, fertility is a major driver of profitability; fertility levels can make the difference between a profitable and non-profitable enterprise. Despite the importance of fertility in tropical beef cattle, relatively little is known about the actual genes which contribute toward the genetic variation of the trait.

The FAANG (Functional Annotation of Animal Genomes) data types aim to characterize genes within the genomes of important animal species and breeds, eventually leading to an understanding of how genetic variation translates to phenotypic variation. FAANG data include but are not limited to 1) RNA-seq (Ozsolak and Milos, 2011), which uses short-read sequencing to quantify gene expression, as well as providing some information on gene structure through the presence of intron spanning reads (Wang Z. et al., 2009); 2) ISO-seq (Gonzalez-Garay, 2016), which uses long-read sequencing technology to identify isoforms and provide some information on gene abundance; and 3) CAGE-seq (cap analysis gene expression sequencing), which uses short-read sequencing and captures the 5′ end guanosine caps of eukaryotic mRNAs to identify transcription start sites (Takahashi et al., 2012a; Takahashi et al., 2012b; Salavati et al., 2020). Through the use of these technologies in combination, it is possible to characterize the structure and abundance of genes and identify potential mechanisms, such as allele-specific expression, that can lead to biological diversity.

A number of genes relevant to Brahman fertility have been identified using genome-wide association studies (Fortes et al., 2012a; Minten et al., 2013; Mota et al., 2017; Müller et al., 2017) and expression studies (Beerda et al., 2008; Moore et al., 2016; Dias et al., 2017; Nguyen et al., 2017; Nguyen et al., 2018; Nguyen et al., 2019). But within the Brahman breed, very little work has been conducted to characterize these critical genes. Here, we apply data from three gene expression datasets generated from a Brahman cow and fetus obtained from the same animal to characterize the transcriptional variation present within these genes within the Brahman breed of cattle.

## Materials and Methods

### Overview

In this study, genes associated with fertility traits in the literature were characterized in two Brahman cattle. Gene sequences from *Bos taurus* annotated genome (Rosen et al., 2020) were located within the Brahman genome (Ross et al., 2022) using BLASTn. CAGE-seq, ISO-seq, and RNA-seq data from 10 different tissues were mapped to these genes. The tissues used were from the same animal as was used to generate the Brahman genome assembly, and tissues from a female fetus she was carrying at the time of slaughter were also taken. The expression characteristics from each of these datatypes were then examined to characterize the expression variation within each gene.

### Sample Collection

The tissues were obtained post commercial slaughter of an Australian Brahman cow from a commercial abattoir. Spleen, longissiumus dorsi muscle, thyroid, ovary, kidney, uterus, lungs, blood, and liver tissues were obtained from the Brahman cow, and lung and liver tissues were obtained from its developing fetus. The tissue samples were collected post commercial slaughter and immediately snap-frozen in liquid nitrogen. The samples were transferred on dry ice and then stored at −80°C until processing.

### Locating Genes in Brahman Genome

Genes that have been previously associated with fertility traits in cattle were identified in the literature. Evidence for an association between the gene and variation in fertility traits included close proximity to a genome-wide association peak or a significant result in a differential expression RNA analysis and other public information, such as a fertility-related phenotype in other species (Table 1).

**TABLE 1:** Genes (identified from literature) as involved in Brahman fertility.

Full Gene Name (Abbreviate gene name)	Function	Evidence for association with fertility in Brahman
Androgen receptor (*AR*)	Member of nuclear receptor superfamily (Fortes et al., 2012b); regulates male fertility *via* regulation of androgen (Brinkmann et al., 1999; Wang R.-S. et al., 2009); interruption of gene function may cause spermatogenesis impairment; feminine character development in males (humans); cancers of prostate, ovary, or breast (MacLean et al., 1995; Dowsing et al., 1999).	Positional candidate in GWAS affecting male and female traits (Lyons et al., 2014).
Insulin-like growth factor 1 (*IGF1*)	Aids in cell growth, differentiation, and transformation (Le Reith, 1997; Yilmaz et al., 2006), regulates release of GnRH which affects age of onset of puberty, conception rate, and maintenance of pregnancy in mammals (Simmen et al., 1993; Wilson, 1998; Velazquez et al., 2008); *IGF1* concentration positively regulates scrotal circumference, motile sperm quantity, and calving rate (Yilmaz et al., 2004; Fortes et al., 2012b).	Positional candidate affecting male and female traits in GWAS including related cattle breeds (Fortes et al., 2013a; Mota et al., 2017).
Inhibin subunit alpha (*INHA*)	Codes for α subunit of inhibin protein (Stelzer et al., 2016); protein complex with α and β subunit negatively regulates secretion of FSH (Richards and Pangas, 2010; Kaneko, 2016; Ulloa-Aguirre et al., 2018); regulates variability of inhibin before puberty (Fortes et al., 2012b).	Candidate gene for GWAS (Fortes et al., 2012b) in males and secretion related to ovulation in females (Burns et al., 2005).
Proenkephalin (*PENK*)	Codes for the neurotransmitters methionine enkephalin and leucine enkephalin *via* proteolytic cleavage (Takahashi, 2016); negatively regulates GnRH *via* modulation of progesterone release (Taylor et al., 2007); negatively regulates LH expression (Malven, 1995)	Biological candidate for GWAS affecting male and female traits (Fortes et al., 2012b) and SNP detection through RNA-seq study (Dias et al., 2017).
Pleiomorphic adenoma gene 1 (*PLAG1*)	Family of zinc finger transcription factors (Juma et al., 2016) influences the stature of cattle which negatively affects fertility (Karim et al., 2011), height of hip, food intake, calving ease, weight at birth, further weight gain, and body size (Snelling et al., 2010; Karim et al., 2011; Pausch et al., 2011; Littlejohn et al., 2012; Fortes et al., 2013b; Juma et al., 2016; Bouwman et al., 2018).	Haplotype analysis (Utsunomiya et al., 2017) and candidate gene for GWAS affecting male and female traits (Littlejohn et al., 2012; Fortes et al., 2013b; Mota et al., 2017).
Ribosomal protein S20 (*RPS20*)	Family of S10P ribosomal proteins (O’Leary et al., 2016) pleiotropically affects body size (McGowan et al., 2008); candidate gene for calving ease and puberty (Pausch et al., 2011; Fortes et al., 2012a).	Functional candidate for GWAS of female traits (Fortes et al., 2012a; Fortes et al., 2013b).
Rhotekin (*RTKN2*)	Family of rhotekin (Collier et al., 2004); codes for rhotekin protein, a part of Rho-binding domain group of Rho-GTPase effectors (Fu et al., 2000), influences exocytosis of the pituitary gland that produces GnRH which regulated the release of LH and FSH (Marques et al., 2000; Momboisse et al., 2011); GWAS study suggests that genetic variation in GnRH stimulation might be influenced by the *RTKN2* gene (Fortes et al., 2012b)	Functional candidate for GWAS of male traits (Fortes et al., 2012b).
Serine peptidase inhibitor, clade A member 7 (*SERPINA7*)	Family of SERPIN (O’Leary et al., 2016) codes for serum thyroid hormone precursor, thyroglobulin (Nonneman et al., 2005) indirectly influences steroid hormone production and spermatogenesis (Nonneman et al., 2005), indirectly affects sex hormone binding globulin level that affects ovarian function (Poppe et al., 2008), and influences the size of testis and sperm count and volume in boars (Dongren et al., 2006; Wagner et al., 2008; Fortes et al., 2012b).	Candidate gene for GWAS in male traits (Fortes et al., 2012b).
SRY-transcription factor 9 (*SOX9*)	Family of the SOX gene (Thomsen et al., 2008) affects male fertility (Thomsen et al., 2008), influences differentiation of sertoli cells in testes, neural crest cells, and chondrocytes (Thomsen et al., 2008), and is associated with testis development. Prostate development and sex determination and sex reversal of embryo (Kent et al., 1996; Vidal et al., 2001; Thomsen et al., 2008; Alankarage et al., 2016).	Candidate gene for GWAS in male traits (Soares et al., 2017).
Signal transducer and activator of transcription 3 (*STAT3*)	Family of STAT protein involved in the growth hormone receptor (GHR) signaling pathway regulating growth hormone or somatotropin, involved in estrogen receptor pathway, and functional disruption causes infertility, obesity, hyperphagia, and thermal dysregulation (Gao et al., 2004).	Candidate gene for GWAS (Mota et al., 2017) and differential gene expression study (Nguyen et al., 2017) for female traits.
Serine/threonine kinase 11 interacting protein (*STK11IP*)	Affects male fertility (Fortes et al., 2012b), affects spermatogenesis in humans, is associated with inhibin expression in Brahman bulls (Fortes et al., 2012b), and affects spermatozoa development related to histone binding (Steilmann et al., 2011).	Functional candidate for GWAS of male traits (Fortes et al., 2012b).
TATA-box binding protein associated factor 1 (*TAF1*)	Family of TBP-associated factors, TAFs (Freiman et al., 2001), part of TFIID, a basal transcription factor (Freiman et al., 2001), involved in development of normal follicles in the ovary (Freiman et al., 2001), influences regulation of cell cycle and differentiation of spermatids in *Drosophila* (Metcalf and Wassarman, 2007), and affects cattle puberty, time of fertilization, and embryo development (Hecht et al., 2011).	Positional and functional candidate for GWAS of male traits (Fortes et al., 2012a).
TATA-box binding protein associated factor 9b (*TAF9B*)	Family of TBP-associated factors, TAFs (Freiman et al., 2001), functions similar to TAF1 and affects scrotal development in Brahman bulls (Fortes et al., 2012a).	Positional and functional candidate for GWAS of male traits (Fortes et al., 2012a).

The coding sequence (CDS) of each target gene was downloaded from the *Bos taurus* gene database of National Centre for Biotechnology Information (NCBI) (Rosen et al., 2020). *Bos taurus* gene sequences were aligned to the genome assembly of the Brahman animal (Ross et al., 2022) using BLASTn (Zhang et al., 2000; National Center for Biotechnology Information, 2008), with an e-value cutoff of 10^−10^.

### Short-Read Whole Genome Sequencing

To identify and confirm the genomic sequence of the genes and to identify heterozygous loci, short-read data from both the Brahman cow and the fetus that were sequenced on the Novaseq6000 S4 flow-cell on a 2 × 150 bp paired-end run were aligned to the Brahman genome assembly (Ross et al., 2022).

To remove low-quality data and adapters, the sequences were quality-trimmed before analysis. The raw sequence data were quality-trimmed using the program QUADTrim (Robinson and Ross, 2019) using the options “*-m 10*” to direct QUADTrim to perform quality trim and N-base filter; “*-g*” to remove the guanosine tail, an error that often results from the optics of the NovaSeq6000; and “-*d bulls*” to specify the pre-set trimming filters specified in the 1000 Bull Genome Project (Hayes and Daetwyler, 2019).

Additionally, to identify genome-wide SNP, the 1000 Bull genomes pipeline (23) was followed. Briefly, reads were aligned to the ARS1.2 Bos taurus genome assembly. Alignments were quality-filtered, and SNPs were called. Loci that were called as the homozygous reference (*Bos taurus*) were removed. SNPs which were homozygous alternative (Ho) and heterozygous (He) were counted. Using the assumption that the whole genome contained 2.7 Gbp, the percentage divergence (D) between the whole genome at the haplotype level was calculated as:

$$D=\frac{\left(\;Ho+\frac{He}{2}\;\right)}{2,700,000,000\;}$$

### RNA Extraction

Total RNA was isolated using mirVana miRNA Isolation Kit (Ambion) following the manufacturer’s instruction. RNA purity was evaluated with a Nanodrop ND-1000 spectrophotometer (v.3.5.2, Thermo Fisher Scientific). QubitTM 4.0 Fluorometer with the Qubit RNA BR (broad-range) assay kit (Thermo Fisher Scientific) was used to quantify RNA concentration. The assessment of RNA integrity was performed using Agilent 2100 Bioanalyser (Agilent Technologies). Only RNA with integrity number greater than 8.0 was used for library preparation for RNA-seq, CAGE-seq, and ISO-seq.

### RNA Sequencing

All RNA samples were sent to the Ramaciotti Centre for Genomics (UNSW Sydney, Australia) for library preparation and sequencing. Stranded paired-end RNA-seq libraries were sequenced on a 2 × 100 bp paired-end NovaSeq6000 run with an S4 flowcell. The resulting reads were trimmed in the same way as the whole genome sequencing Illumina data.

### CAGE-seq Sequencing

The RNA was sequenced on Illumina single-read flow cells utilizing the 27-nt-long tags prepared corresponding to the 5′-end of the capped RNAs as per Salavati et al., (2020). The libraries were sequenced on an Illumina HiSeq 2500 platform (50 nt single-read) at the Centre for Genomic Research, University of Liverpool, Liverpool. After sequencing, read quality was assessed using FastQC (Andrews, 2010), and quality trimming was administered using Trimmomatic, version 0.35 (Bolger et al., 2014) using the settings “*CROP:9*” to trim the last nine bases and “*HEADCROP:14*” to trim the initial 14 base pairs (Forutan et al., 2021).

### ISO-seq Sequencing

First-strand cDNA synthesis was conducted using the TeloPrime Full-Length cDNA Amplification kit (Lexogen, Australia) from 1 μg of total RNA input according to the manufacturer’s guideline, with an exemption of the uterus and ovary samples. Due to low extracted RNA concentration, only 500 ng of total RNA from these two tissues was used at this step. Additionally, among these tissue samples, the full-length double-stranded cDNAs from the fetal liver, thyroid, and spleen were prepared using the TeloPrime Full-Length cDNA Amplification version 1 kit (Lexogen, Australia). The TeloPrime Full-Length cDNA Amplification version 2 kit was used for all other samples.

To determine the optimal PCR cycle number for the large-scale PCR, the full-length double-stranded cDNAs were fist amplified in a qPCR reaction using 3′ and 5′ end-specific primers from the TeloPrime Full-Length cDNA Amplification kit (Lexogen, Australia) combined with PCR master mix reagents from PrimerSTAR GXL DNA Polymerase (Takara, Australia). SYBR Green I (Invitrogen, United States) was added to a final concentration of ×0.1 in the qPCR reaction with a total of 40 cycles. QPCR results were evaluated based on the fluorescence value, electrophoresis images, and bioanalyzer results using the Agilent DNA 12000 kit (Agilent Technologies, Germany). Large-scale PCR was then performed using optimal PCR cycles determined during the optimization step for each sample.

PCR products for all samples were sent to Ramaciotti Centre for Genomics (UNSW Sydney, Australia) for library preparation and sequencing. Briefly, PacBio IsoSeq libraries were prepared using the PacBio SMRTBell template prep kit 1.0 SPv3 for sequel protocol. Aliquots of the cDNA products underwent a ×1 and ×0.4 Ampure PB clean-up (Beckman Coulter, Australia). The aliquots were combined post clean-up using different ratios with preference to the aliquots enriched for transcripts above 4 kb. The libraries were sequenced on the PacBio Sequel system using 10-hour movies and v3.0 sequencing chemistry. A total of 11 SMRT cells were sequenced on a PacBio Sequel system (Ramaciotti Centre for Genomics, UNSW Sydney, Australia) for 10 samples. The fetal lung sample was sequenced twice as the first run was overloaded.

Demultiplexing, filtering, and quality control were performed using SMRT Link version 6.0.0 (Pacific Biosciences). The raw reads (subreads) generated by PacBio were used for calling circular consensus sequence (CCS) using the CCS tool (version 4.2.2) with parameters “—skip-polish –min-passess=3.” Adapter sequences from these CCS reads were removed using Lima tool (version 1.11.0) with parameters “lima –isoseq –dumclips”. The polyA tails and artificial concatemers were trimmed and removed using the refine tool (isoseq refine –require-polya –min-length-polya 8).

### Mapping of Expression Data

All three datasets (CAGE-seq, RNA-seq, and ISO-seq) used in this study and the gene sequences were mapped to the Brahman genome that was assembled from the same animal (cow) as all the nonfetal expression data were generated from.

The CAGE-seq data were mapped using BWA-mem (Li and Durbin, 2009) optimized as per Forutan et al. (2021) with the options -M (to mark the shorter split hits as secondary hits), -k 10 (to specify that the sequences with seed length below 10 were skipped), -T 10 (to filter out alignments with a mapping score less than 10), -L 4 (to specify the clipping penalty), and -B 5 (to specify the mismatch penalty).

RNA-seq data were mapped to the Brahman genome using STAR (Dobin et al., 2013). The reference genome was indexed using the “genomeGenerate” option. The alignments were output in sorted BAM format.

Minimap2 (Li, 2018; 2019) was used to align the gene sequences and ISO-seq data. The output was in “SAM” format, and the options “-uf” was used to find canonical splicing sites GT-AG on the transcript strand, “--secondary =no” to skip the output of secondary alignments, and “ -C5 -O6,24 -B4” which is the pre-set filter for long-read splice alignment of PacBio circular consensus sequencing reads. The alignments were converted to a sorted bam file using Samtools (Li et al., 2009).

Integrative Genome Viewer (IGV) (Robinson et al., 2011) was used to visualize alignments to interpret transcription start sites, isoforms, and gene expression. The minimum mapping quality was set to 10.

### Identifying Heterozygous Loci

The heterozygous loci were identified using whole genome sequencing of the same animals. Single-nucleotide polymorphisms (SNPs) were observed manually in IGV. SNPs were only considered where both alleles were observed in at least two reads.

### Statistical Analysis

To calculate the statistical significance of allele-specific expression for each tissue in the RNA-seq data, Pearson’s chi-squared test (LaMorte, 2016) was administered by comparing the difference between the expected and observed value with df = 1. The formula is as follows:

For each allele i,

$$\chi_{df=1}^{2}=\sum \frac{{\left(O_{i}-E_{i}\right)}^{2}}{E_{i}}\;$$

where O_i_ is the observed value and E_i_ is the expected value for each of the two alleles.

Only loci that were heterozygous, based on the observation of both alleles in the whole genome sequence data, were tested. The ratio of the two alleles at each of the heterozygous loci tested was assumed to be 1:1. Hence, the E-value is calculated by taking the average of the observed value. Therefore,

$$E_{i}=\;\frac{\text{R}}{2}$$

where E_*i*_ is the expected value for the allele *i* and R is the total number of reads at that locus in the RNA-seq data (sequencing depth).

To control for any sequencing bias between the two alleles, instead of assuming equal allelic ratios, the ratio of alleles observed in the whole genome sequencing data from each of the two animals was also used to determine the expected values. Hence, the expected value for each allele *i* was

$$E_{i}=\;\frac{\text{R}\;\times G_{i}}{\sum G}$$

where E_*i*_ is the expected value for the allele *i*, R is the total observations at that locus in the RNA-seq data (sequencing depth), G_*i*_ is the observations of allele i in the whole genome sequencing (WGS) data, and ∑G is the total number of observations in the WGS data at that locus (sequencing depth).

To compare gene expression between tissues, the number of reads from each dataset (RNA-seq, ISO-seq, and CAGE-seq) was used. The relative expression in reads per million for each gene in each tissue (RPM_*ij*_) was calculated as

$$\mathrm{RP}M_{\mathrm{ij}}=T_{\mathrm{ij}}÷A_{j}\times 1,000,000$$

where T_*i*_ is the total number of read pairs mapped to gene *i* in tissue *j* and A _*j*_ is the total read pairs mapped in tissue *j*.

## Results

### Identification of Important Fertility Genes in the Brahman Genome

Thirteen genes important for Brahman fertility were identified in the literature (Table 1). The CDS sequence was extracted from the *Bos taurus* genome and aligned to the Brahman genome assembly using BLASTn to obtain the positions of those genes within the Brahman genome (Table 2). The mean homology of the 13 genes between the *Bos taurus* and Brahman genome was 99.76%. The mean homology across the entire genome of the two individuals was 99.50% (Table 3). Five genes had at least one isoform that shared a 100% homology between *Bos taurus* and *Brahman* genomes. All isoforms of proenkephalin (*PENK*), pleiomorphic adenoma gene 1 (*PLAG1*), SRY-transcription factor 9 (*SOX9*) and TATA-box binding protein-associated factor 9b (*TAF9B*), and three out of nine isoforms in insulin-like growth factor 1 (*IGF1*) had 100% homology. The least homology was observed in the three isoforms of rhotekin (*RTKN2*) ranging from 99.26 to 99.33% (Table 2).

**TABLE 2:** List of genes with their respective positions within *Bos taurus* genome and Brahman genome with their alignment length and identity.

Gene	Isoforms	Bos taurus		Bos indicus			Length of gene^a^	Chromosome	Strand direction^b^	Homology (%)
		Start Position	Stop Position	Start Position	Stop Position					
AR	X1	51674157	51881942	84786064	84957236	171172		X	Negative	99.96
IGF	X1	66206081	66263849	66145894	66203733	57839		5	Negative	100
	X2	66206081	66263849	66145894	66203733	57839				99.65
	X3	66206081	66261980	66145894	66199198	53304				100
	X4	66206081	66261980	66145894	66199195	53301				100
	X5	66192424	66263849	66132237	66203733	71496				99.37
	X6	66192595	66263849	66132408	66147706	15298				99.33
	X7	66192424	66261980	66132237	66147706	15469				99.76
	X8	66192424	66261980	66132237	66147706	15469				99.75
	Preprotein	66192424	66263849	66132237	66147706	15469				99.79
INHA	X1	107501844	107504762	107614722	107617642	2920		2	Positive	99.82
PENK	X1	23542677	23546157	23420291	23423774	3483		14	Negative	100
PLAG1	X1	23330541	23332546	23192090	23194095	2005		14	Negative	100
	X2	23330541	23331794	23192090	23193343	1253				
RTKN2	X1	18284769	18393694	17858619	17967072	108453		28	Negative	99.3
	X2	18284769	18385527	17858619	17958855	100236				99.26
	X3	18284769	18348138	17858619	17921964	63345				99.33
SERPINA7	X1	54824445	54829800	53237946	53241468	3522		X	Positive	99.69
	Precursor	54824445	54827949	53237963	53241468	3505				99.68
SOX9	X1	58919579	58922699	60166722	60169843	3121		19	Negative	100
STAT3	X1	42419849	42450618	43645371	43676141	30770		19	Negative	99.66
	X2	42419849	42450618	43645371	43676141	30770				99.66
	X3	42421282	42450618	43647176	43676141	28965				99.64
STK11IP	X1	107521189	107535476	107634090	107648385	14295		2	Positive	99.45
	X2	107521189	107535476	107634090	107648385	11551				99.5
	X3	107521189	107533477	107634090	107646987	12297				99.46
TAF1	X1	79206804	79276760	80853773	80923837	70064		X	Negative	99.86
	X2	79206804	79276760	80853773	80923837	70064				99.86
	X3	79206804	79276760	80853773	80923837	70064				99.79
	X4	79206804	79276760	80853773	80923837	70064				99.84
	X5	79206804	79276760	80853773	80923837	70064				99.84
	X6	79197983	79276760	80857229	80923837	66608				99.87
	X7	79225602	79276760	80872738	80923837	51099				99.9
	X8	79229398	79276760	80885675	80923837	38162				99.9
	X9	79227390	79276760	80885675	80923837	38162				99.9
TAF9B	X1	74232003	74255254	74070058	74093312	23254		X	Positive	100
	X2	74232003	74255924	74070058	74093982	23924				100
	Subunit	74232003	74239263	74070058	74077335	7277				100

a Length from the first base of the first exon to the last base of the last exon.

b Negative = 3′ to 5′ direction; positive = 5′ to 3′ direction.

**TABLE 3:** Genome-wide SNPs compared to *Bos taurus* genome.

	Mother	Foetus
Homozygous SNP^a^	7762707	7734830
Heterozygous SNP	11408849	11537221
Haplotype level Homology	99.501%	99.500%

a Homozygous for alternate alleles from reference.

Within the 13 genes, heterozygous sites were identified in both the Brahman cow and fetus. There were 270 and 95 SNPs identified within the coding regions of the fertility genes for the cow and fetus, respectively. 55.07% of these were located within the untranslated regions (3.01% in 5′ UTR and 52.05% in the 3′ UTR). The highest number of heterozygous sites was observed in *STK11IP*; no significant heterozygous sites were observed in *TAF1*.

### Transcription Start Sites

The transcription start sites (TSSs) were identified using CAGE-seq data (Supplementary Table S1) from Forutan et al. (2021), which were remapped to the Brahman genome (Ross et al., 2022). No CAGE-seq peak was identified in the *PLAG1* region. In the 12 out of 13 genes that had a TSS identified, the CAGE-seq peaks were on an average 562 base pairs upstream of the start of the coding region in the first exon in the 5′ direction (Table 4). The largest 5′ UTR was found on *SERPINA7,* and the smallest was in *TAF9B*.

**TABLE 4:** Position and length of 5′ untranslated region.

Gene	5′ gene position	CAGE-seq TSS peak		UTR length^a^
		Start position	Stop position	
AR	84957236	84958298	84958477	1,241
IGF1	66203733	66203715	66204034	301
INHA	107614722	107614985	107614491	231
PENK	23423774	23424308	23424459	685
RPS20	23140852	23141036	23141219	367
RTKN2	17967072	17966877	17967512	440
SERPINA7	53237946	53236117	53236027	1,919
SOX9	60169843	60169524	60170244	401
STAT3	43676141	43676023	43676320	179
STK11IP	107634090	107634194	107633817	273
TAF1	80923837	80923816	80923981	144
TAF9B	74070058	74070080	74069977	81

a Distance between the start of the coding region within the first exon and the center of the TSS peak from the CAGE-seq data.

### Tissue-Specific Expression

**FIGURE 1:**
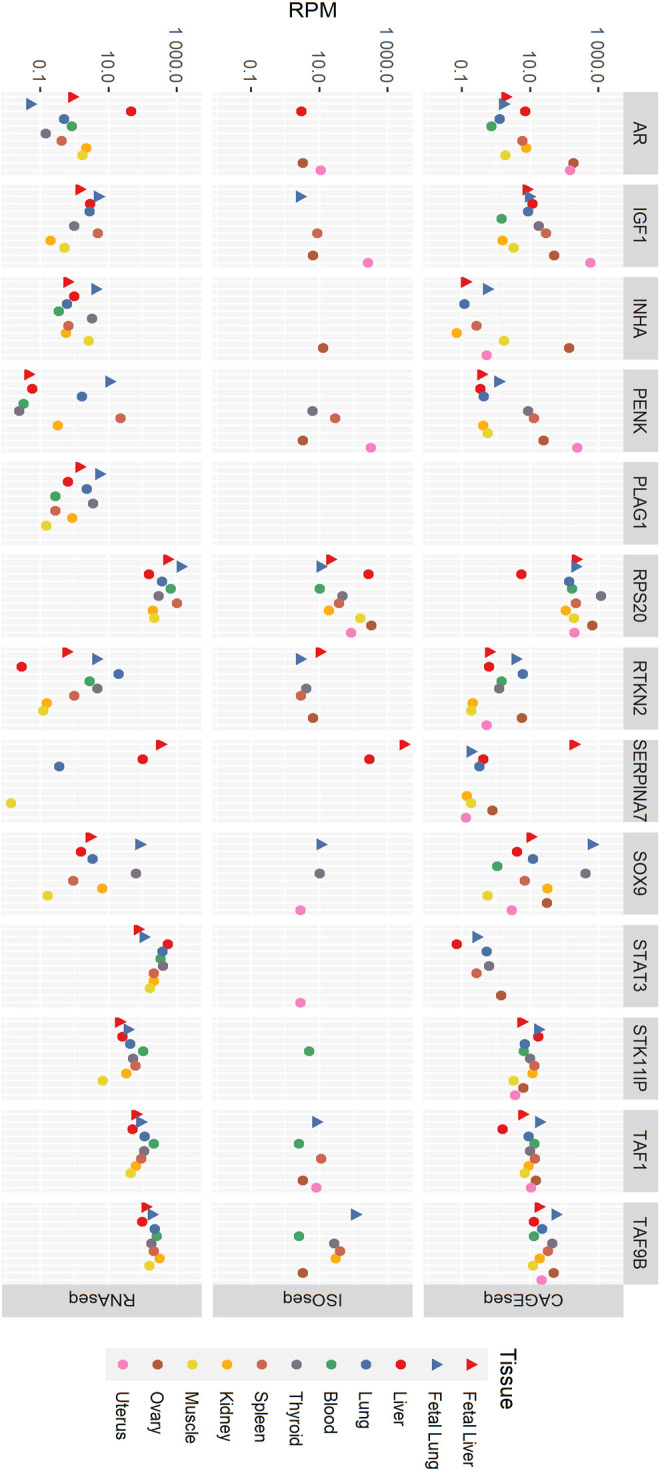
Tissue-specific expression of genes in RNA-seq, ISO-seq, and CAGE-seq data. RPM, reads per million.

Tissue-specific expression of the 13 fertility-related genes was examined by comparing reads per million in all three datasets. In RNA-seq data (Figure 1; Supplementary Table S1) in blood, *RPS20* has the most expression followed by *STAT3*, *TAF9B,* and *TAF1. IGF1*, *SERPINA7,* and *SOX9* were not expressed at detectable levels in the blood sample, and the expression of the rest of the genes was very low. *RPS20* was the most expressed gene in fetal liver followed by *SERPINA7* and the least being *PENK*, *RTKN2,* and *INHA* with rest of the gene expression being insignificant. In adult liver tissue, *STAT3* was the most prominently expressed gene followed by *RPS20*. While other genes showed a relatively similar expression profile between the two liver life stages, the expression level of *AR* was much higher in liver tissue than that in fetal liver. *STAT3* was the most expressed gene in thyroid followed by *RPS20*. The expression of genes *RTKN2*, *STAT3*, *STK11IP*, *TAF1,* and *TAF9B* was higher in adult lung tissue than that in fetal lung, whereas for genes *IGF1*, *INHA*, *PENK*, *PLAG1*, *RPS20,* and *SOX9,* fetal lung showed higher expression. *AR* and *SERPINA7* had no significant expression in both these tissues. *TAF9B* was the most expressed in kidney tissue, while *TAF1* expression is considerably lower. *STAT3* and *RPS20* also had significant expression in kidney tissue. Most genes were lowly expressed in muscle tissue with an exception of *RPS20*, *STAT3*, and *TAF9B*. The spleen and fetal lung showed the highest expression level of *RPS20*, followed by *STAT3* and *TAF9B*, when other genes have considerably lower expression levels. The fetal lung expresses *SOX9* relatively highly, while its expression in the spleen was very low. Overall, fetal lung and spleen had the highest expression across all of the tested genes in the RNA-seq data.

In the CAGE-seq data (Figure 1; Supplementary Table S1), *RPS20* was the most expressed gene with its highest expression in the fetal lung and ovary. *STK11IP* and *TAF9B* also shows significant expression in all tissues. *PLAG1* had no CAGE-seq expression data, and *STAT3* shows no significant expression in any of the tissues. *SERPINA7* and *INHA* showed significant expression in only the fetal liver and ovary, respectively. *IGF1* was most expressed in the uterus along with significant expression in the liver and lung tissues, ovary, spleen, and thyroid. *AR* showed high expression levels in the ovary and uterus as well as the kidney, liver, and spleen. *SOX9* has a fairly high expression in all tissues except blood and muscle, while *TAF1* is well expressed in all tissues except the adult liver. *PENK* is highly expressed in the uterus in addition with notable expression in the ovary, spleen, and fetal lung.

In the ISO-seq data (Figure 1; Supplementary Tables S1, S2), *RPS20* was the only gene with detected expression in all tissues, while *PLAG1* showed no expression in any tissue. The ovary and uterus were the tissues where the most genes were expressed despite not having the highest sequencing depth. The ISO-seq data had the lowest sequencing depth of the three technologies used. Given the lower sequencing depth of the ISO-seq data, it is very likely that there are many more uncharacterized isoforms in the data; this is a limitation of this study.

In the ISO-seq data, genes including *PLAG1*, *SERPINA7*, *STAT3*, *SOX9*, and *STK11IP* were highly expressed in the ovary, while *AR*, *IGF1*, *PENK*, *RPS20*, *STAT3*, *SOX9*, and *TAF1* were highly expressed in the uterus. Most genes were not expressed in the muscle and kidney. Only *RPS20* was expressed in muscle, and only *RPS20* and *TAF9B* were expressed in the kidney. *TAF9B* was expressed in the blood, fetal lung, kidney, ovary, spleen, and thyroid. *RTKN2* shows expression in the fetal liver but not in the adult liver. It is also expressed in the fetal lung, ovary, spleen, and thyroid. *INHA* is only expressed in the ovary, *STAT3* was only observed in the uterus and *STK11IP* only in blood. Despite *SERPINA7* being expressed only in the liver tissues, it was the most highly expressed gene in the ISO-seq dataset with its highest expression level in the fetal liver. *AR* was expressed in the adult liver, ovary, and uterus and *SOX9* is expressed in the fetal lung, thyroid, and uterus. *IGF1* was expressed in the ovary, spleen, thyroid, and uterus. The highest expression of *PENK* is found in the uterus, while it is also expressed in the fetal lung, ovary, and spleen at lower levels.

Overall, *RPS20* was the most highly expressed gene in any tissue in the RNA-seq, ISO-seq and CAGE-seq data. When the expression level was corrected by length to reads per kilobase million (by dividing by the length of the coding sequence for each gene in kilobasepairs), *RPS20* was still the most highly expressed gene in the RNA-seq data, consistent with result in the CAGE-seq data.

### Allele-Specific Expression

**FIGURE 2:**
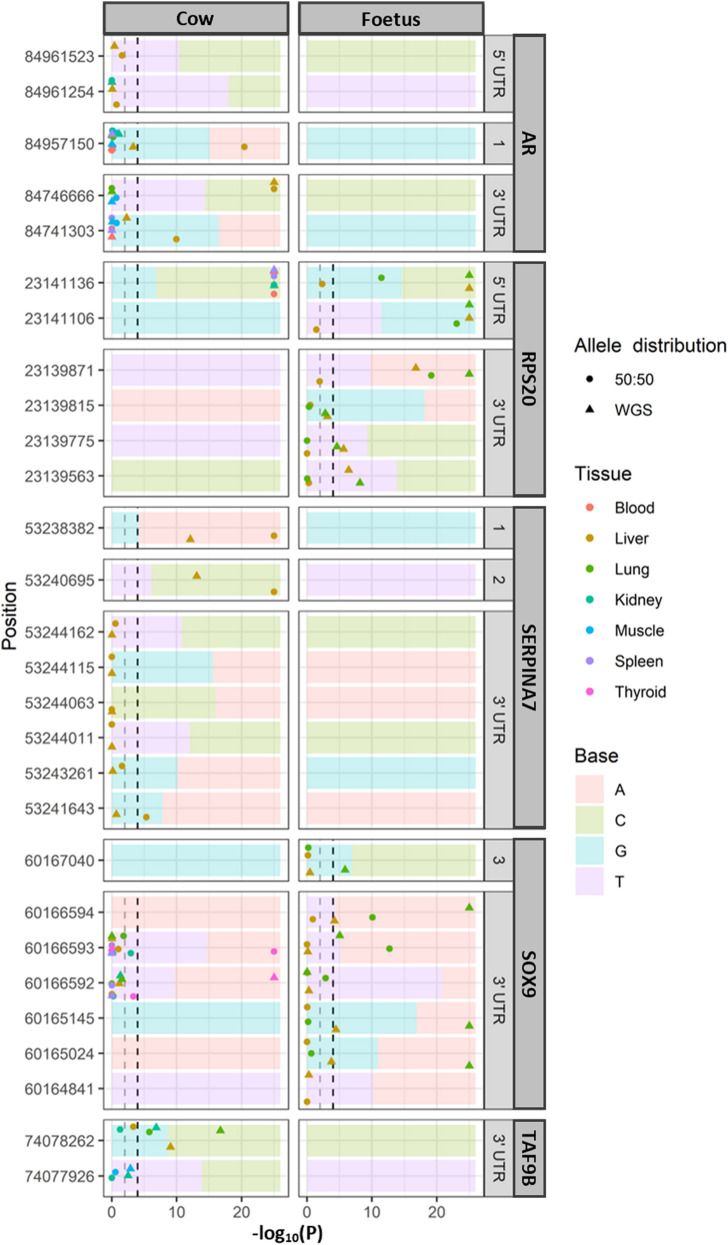
Allele-specific expression for each tissue with significance. Significance at *p* < .0001 and *p* < .01 indicated for reference (dashed lines). The ratio of alleles observed in whole genome sequencing (WGS) of the same animals is indicated as the background of each panel. Only loci that were heterozygous in the WGS data were tested for allele-specific expression. In the position, 1, 2, and 3 refer to exons 1, 2, and 3, respectively.

Allele-specific expression (Figure 2) was tested where there were expression data which overlapped a heterozygous SNP in the relevant animals (the cow or the fetal sample). A total of 117 SNPs were observed in the all genes. *TAF1* had no SNP identified in these two animals. Due to the large number of loci being tested, a *p*-value cutoff of .0001 was used. Out of the 117 SNPs tested, nine had allele-specific expression in the cow and five in the fetus (chi-squared test; *p* < .0001). To determine the significance of these SNPs was not an effect of the allelic bias in the RNA-seq data; the expected ratios were adjusted to reflect the allelic ratios in the WGS data. In the cow, significant allele-specific expression was still observed in *AR*, *RPS20*, *SERPINA7*, and *TAF9B* (chi-squared test; *p* < .01). The SNP in *SOX9* was not significant when using the new WGS-based ratio. Within the fetal tissues, *RPS20* and *SOX9* showed allele-specific expression when compared to both the 50:50 ratio and WGS-based expected ratios. Of the cow tissues, only the liver displayed allele-specific expression of *AR*, while all tested tissues showed highly significant allele-specific expression in the 5′ UTR of RPS20, which were the fetal tissues. *SERPINA7* showed allele-specific expression in exons 1 and 2 in the liver tissue of the cow, which was the only sample with sufficient coverage to test for ASE. The 3′ UTR of *SOX9* and *TAF9B* had allele-specific expression in both the fetal lung and adult lung. Overall, more SNPs associated with ASE were identified in the 5′ and 3′ UTR of the investigated genes than the exons.

**FIGURE 3:**
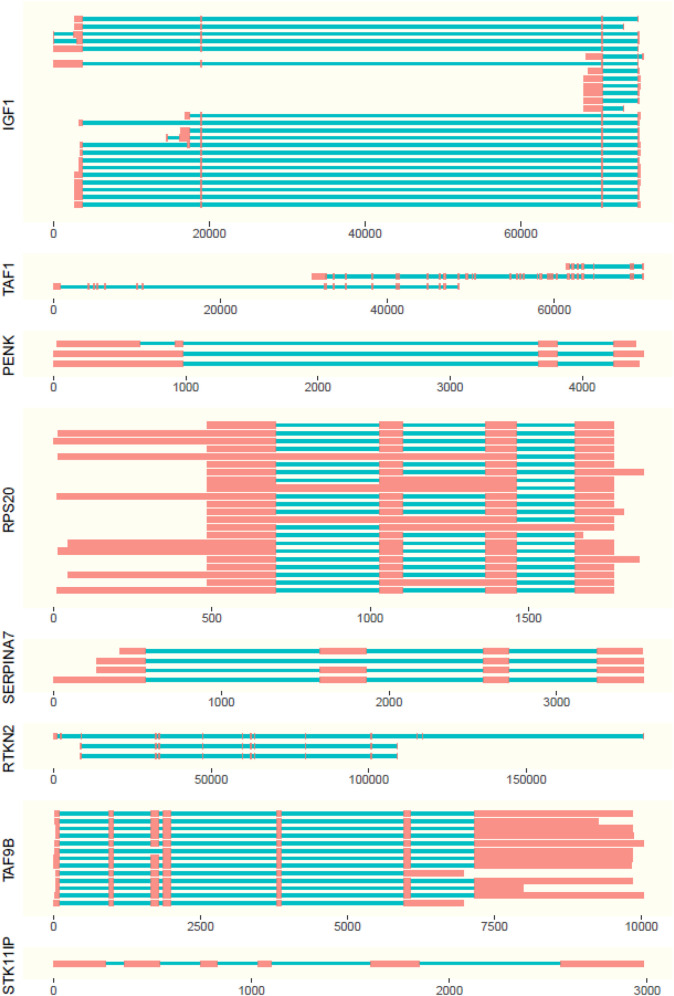
Representation of isoforms with length of exons (red boxes) and introns (blue lines). Positions are displayed relative to the first expressed base pair (x axis) of the gene.

### Isoform Discovery

The isoforms present in the genes were observed in the ISO-seq data with supporting evidence from RNA-seq data (Figure 3; Supplementary Tables S3–S6). For the scope of this study, the isoforms absent in the NCBI database are considered to be novel.

Out of all examined genes, the following had no ISO-seq data mapping to the gene region: *AR*, *PLAG1*, *SOX9*, and *STAT3*. The genes *PENK* and *INHA* had only one isoform each, which were not novel. *STK11IP* had one isoform expressed in blood, but it was not well supported with evidence from RNA-seq.

The highest number of isoforms was observed in *SERPINA7* with seven isoforms expressed in adult and fetal liver tissues. Out of these isoforms, isoforms I, II, IV, V, VI, and VII were novel (Figure 3). Genes *RPS20* and *IGF1* (Figure 3) have four isoforms each. Isoform 1 of *RPS20* is present in the fetal liver, kidney, liver, muscle, ovary, spleen, and uterus, while isoform III is present in the kidney and IV in the liver. Isoform II is present in all tissues. Out of these isoforms I, II, and II are found to be novel. In *IGF1*, isoform I is found in the ovary and uterus, while isoform II is there in the spleen and thyroid. Isoforms III and IV, which are both expressed in the uterus, are novel.

The gene *RTKN2* was expressed in the fetal lung, ovary, spleen, and thyroid and has three isoforms out of which all three were novel. Isoform I, with a longer first exon, was found only in the fetal lung and thyroid. Isoform II was observed in the spleen, whereas III was found in the ovary and had the first 2 exons missing (Figure 3).

A novel isoform, which missed the first seven exons when compared to the NCBI database of *TAF1* isoforms, was identified for the *TAF1* gene. It is expressed only in the spleen (Figure 3).

*TAF9B* had two isoforms. The absence of the first exon in 5′ direction when compared to the *Bos taurus* gene sequence made both these isoforms novel. Isoform I was present in the blood and ovary and II was observed in the fetal lung, kidney, spleen, and thyroid (Figure 3).

Interestingly, *TAF9B* was a partially nested gene. The last exon of *TAF9B* was nested in phosphoglycerate kinase 1 in blood, kidney, lung, muscle, thyroid, fetal liver, and fetal lung tissues.

## Discussion

Here, we investigated expression variation of 13 fertility-associated genes in Brahman cattle using RNA-seq, ISO-seq, and CAGE-seq. Within these few genes, the variety of data available allowed us to identify previously unknown levels of variation at the genome and transcriptome level.

The homology between the *Bos taurus* genome annotation and Brahman genome of 99.8% reveals that on average, these genes have a variable site within their expressed regions roughly every 500 bp. This is a slightly higher level of conservation than that of the whole genome, which has 99.5% homology in both the mother and fetus based on identified SNP loci. A higher level of conservation within these genes could be expected given their role in important reproductive traits and the selective pressure in coding or regulatory regions. However, given the extremely small number of animals used in this study, this finding needs to be validated across a large cohort of genetically diverse animals.

Identification of transcription start sites (TSSs) in these genes revealed that (where detected) the TSSs were on an average 562 basepairs upstream of the start of the coding region in the first exon. 5′ UTRs are known to regulate the posttranscriptional modification (Kim et al., 1992; Lawless et al., 2009; Araujo et al., 2012). These play an important role during embryonic development (Van Der Velden and Thomas, 1999; Leppek et al., 2018). 5′ UTRs also regulate the translation of mRNAs (Van Der Velden and Thomas, 1999). Studies suggest that 5′UTR may be utilized to control the expression of genes (Halder et al., 2009). The TSSs within our investigated genes revealed variable 5′ UTR lengths (81–1,919 bp). This is within the observed ranges of 5′ UTR across mammals (Pesole et al., 2001); however, 12 out of the 13 genes had 5′ UTR longer than the reported average for other mammals (nonhuman and nonmouse), while nine out of 13 genes had longer 5′ UTRs than the average reported for humans. Compared to UTR lengths in humans observed from the study by Davuluri et al., (2000), the 5′ UTRs of these genes are substantially longer in most cases (averaging from 47 to 250 bp depending on the expression class). This suggests that these genes related to fertility may have longer than average 5′ UTRs, which may have a role in their regulation (Pesole et al., 2001). Davuluri et al. (2000) found that genes which were thought to be poorly translated tended to have longer 5′ UTRs, suggesting that the translation of these fertility-related genes may be low, especially for *SERINPINA7* and AR, both of which had 5′ UTRs longer than 1KB.

The long 5′ UTR observed in *AR* may lead to low translation. Lyons et al. (2014) suggested that this low level of translation may be overcome by a shift in the transcription start site or alternative splicing. *AR* has a cluster of SNPs and indels in the 5′UTR region in cattle. Within this polymorphism cluster, there are four putative SRY-binding sites in a perfect LD with an SNP associated with scrotal circumference in Brahman bulls (Lyons et al., 2014). Additionally, a knockout study in mice found that *AR* also affects fertility in females (Yeh et al., 2002), suggesting that even though *AR* is primarily involved in male development, it may also contribute to female fertility variation.

This is only the second report of CAGE-seq data analysis in cattle. Here, we used the same dataset as used by Forutan et al. (2021) who reported the structure of TSS positioning within the bovine genome across subspecies and developmental stages. Additionally, a CAGE-seq analysis mapped, identified, and predicted novel and previously unannotated transcription start sites (TSSs) and TSS enhancer cluster (Salavati et al., 2020) in sheep. Here, we took a more specific approach to investigate a subset of genes.

The RNA-seq results revealed that the highest relative expression of these genes is observed in the spleen and fetal lung. This is consistent with the fact that most of these genes considered for this study are related to hormonal regulation and reproductive development (Kent et al., 1996; Le Reith, 1997; Brinkmann et al., 1999; Vidal et al., 2001; Yilmaz et al., 2006; Wang R.-S. et al., 2009). Expression of genes observed in ISO-seq and CAGE-seq data was not as prominent as that in RNA-seq. The sequencing depth within these datasets is much lower than that in RNA-seq, limiting their power to gene expression. It is important to note that a cDNA size selection step was included in the ISO-seq library preparation, which could skew the quantification dramatically. For the CAGE-seq results, the short-read length could limit the ability of the data to map specifically to the genome as there are regions of high homology, such as recent duplications. Therefore, RNA-seq is likely the most accurate representation of relative abundance between the tissues of the three expression datasets used in this study.

The absence of any data for *PLAG1* in ISO-seq and CAGE-seq datasets could be a direct effect of the lack of deep sequencing. Genes such as *AR*, *SERPINA7*, *SOX9*, and *STK11IP* have shown very low expression in all the datasets despite being found to be expressed well in some of these tissues by previous studies (O’Leary et al., 2016). These genes are mainly associated with male fertility, while the available datasets were compiled from a female specimen (both the cow and the fetus were female); this is the most likely cause of the lower expression levels.

Allele-specific expression was identified in five out of the 13 genes. Liver and fetal liver tissues showed the highest proportion of SNPs with allele-specific expression. Allele-specific expression has been found to be abundant in dairy cattle transcriptome data (Chamberlain et al., 2015), and this analysis confirms that at least for these genes, it is likely the same in Brahman cattle also. Allele-specific expression in a direct mechanism where genetic variation can be linked to phenotypic variation for an important trait as the amount of a gene being expressed can be directly affected by which alleles are present in the animal.

A total of 24 isoforms were found within all the observed genes, out of which 16 are novel. Isoform III of *RPS20* shows an unspliced intron in the ovary and uterus. It is also notable that when intron 3 is retained, intron 2 is always retained, but when intron 2 is retained, intron 3 is not necessarily always retained. Unspliced introns are thought to have a major role in gene expression regulation in plants (Pleiss et al., 2007; Syed et al., 2012), but the part they play in mammals is not fully known. Studies suggest that intron retention may regulate the production of isoforms, stability, and efficiency of translation of RNA and rapid gene expression through posttranscriptional splicing of these introns (Jacob and Smith, 2017). Multiple ISO-seq studies in different species (Xie et al., 2018; Beiki et al., 2019; Feng et al., 2019) have identified that intron retention is quite common as a source of isoform variation.

An important limitation of this study is that only two related animals were used. Given the effect that genetic variation has been observed to have of isoform expression (Garrido-Martín et al., 2021), it could be hypothesized that in a larger population of genetically diverse animals, more variation in isoforms would be observed. The economics of data generation using current technology mean that whole population isoform characterization using long-read sequencing is not currently feasible. However, as sequencing technology continues to improve and costs decrease, population scale isoform discover is likely to be a reality in the near future. Large-scale population datasets will likely show dramatically more variation that has been observed here.

An important aspect of isoform discovery *en masse* is that stringent filters must be placed on the data interpretation to ensure that reported isoforms are robust. This generally means that isoform calling pipelines will collapse down isoforms where the only difference is in the first or last exon or the untranslated regions. The isoform variation in *TAF9B,* where only the length of the untranslated region differs between the two isoforms, is one such example. Usually isoforms such as these would raise suspicion of the shorter isoform being 5′ degraded; however, as there are multiple forms of evidence used to identify these isoforms (ISO-seq and RNA-seq) we can be confident that there are, indeed, two gene forms being expressed. This particular example highlights the usefulness of using multiple data types to identify expression variation.

Despite the absence of the last exon in *TAF9B* in the ISO-seq data, RNA-seq data suggest the presence of an exon downstream in 3′ direction in blood, fetal lung, kidney, ovary, spleen, and thyroid tissues. This is confirmed by a large number of intron spanning RNA-seq reads. This suggests the presence of an exon within another gene (phosphoglycerate kinase 1) present right next to *TAF9B* on the reverse strand. The relevance of this exon on the regulation of expression of both *TAF9B* and the other gene needs to be considered. Studies of nested genes, where genes were fully located within other genes (Yu et al., 2005) showed that the nested genes were under strong selection and displayed reciprocal expression with each other as well as strong tissue-specific expression. We did not observe strong tissue-specific expression to *TAF9B*, and the partially-nested gene it is associated with was also expressed in six of the tissues where *TAF9B* was observed, suggesting that *TAF9B* does not follow the same pattern of expression.

Genes relevant to fertility in Brahman were identified and shown to have tissue-specific expression, allele-specific expression, variation in transcription start sites, untranslated regions, and novel isoforms. This case study is an example of the detailed information that can be obtained from combining information from multiple expression datasets. It is clear that no one datatype is able to fully characterize the transcriptome, and efforts to strategically align data generation efforts will be most beneficial.

## Data Availability

CAGE-seq sequencing data are available *via* the European Nucleotide Archive (ENA) under study ID PRJEB44817. The RNA-seq and ISO-seq data for all of the genes presented in the article are available in the Supplementary Material.

## References

[B1] Alankarage D. Lavery R. Svingen T. Kelly S. Ludbrook L. Bagheri-Fam S. (2016). SOX9 Regulates Expression of the Male Fertility Gene Ets Variant Factor 5 ( ETV5 ) during Mammalian Sex Development. Int. J. Biochem. Cell Biol. 79, 41–51. 10.1016/j.biocel.2016.08.005 27498191

[B2] Andrews S. (2010). FastQC: A Quality Control Tool for High Throughput Sequence Data [Online]. Available at: http://www.bioinformatics.babraham.ac.uk/projects/fastqc/ .

[B3] Araujo P. R. Yoon K. Ko D. Smith A. D. Qiao M. Suresh U. (2012). Before it Gets Started: Regulating Translation at the 5' UTR. Comp. Funct. Genomics 2012, 475731. 10.1155/2012/475731 22693426 PMC3368165

[B5] Beerda B. Wyszynska-Koko J. Te Pas M. F. W. De Wit A. A. C. Veerkamp R. F. (2008). Expression Profiles of Genes Regulating Dairy Cow Fertility: Recent Findings, Ongoing Activities and Future Possibilities. Animal 2, 1158–1167. 10.1017/s1751731108002371 22443728

[B6] Beiki H. Liu H. Huang J. Manchanda N. Nonneman D. Smith T. P. L. (2019). Improved Annotation of the Domestic Pig Genome through Integration of Iso-Seq and RNA-Seq Data. BMC Genomics 20, 344. 10.1186/s12864-019-5709-y 31064321 PMC6505119

[B7] Bolger A. M. Lohse M. Usadel B. (2014). Trimmomatic: a Flexible Trimmer for Illumina Sequence Data. Bioinformatics 30, 2114–2120. 10.1093/bioinformatics/btu170 24695404 PMC4103590

[B8] Bouwman A. C. Daetwyler H. D. Chamberlain A. J. Ponce C. H. Sargolzaei M. Schenkel F. S. (2018). Meta-analysis of Genome-wide Association Studies for Cattle Stature Identifies Common Genes that Regulate Body Size in Mammals. Nat. Genet. 50, 362–367. 10.1038/s41588-018-0056-5 29459679

[B9] Brinkmann A. O. Blok L. J. De Ruiter P. E. Doesburg P. Steketee K. Berrevoets C. A. (1999). Mechanisms of Androgen Receptor Activation and Function. J. Steroid Biochem. Mol. Biol. 69, 307–313. 10.1016/s0960-0760(99)00049-7 10419007

[B10] Burns D. S. Jimenez-Krassel F. Ireland J. L. H. Knight P. G. Ireland J. J. (2005). Numbers of Antral Follicles during Follicular Waves in Cattle: Evidence for High Variation Among Animals, Very High Repeatability in Individuals, and an Inverse Association with Serum Follicle-Stimulating Hormone Concentrations1. Biol. Reprod. 73, 54–62. 10.1095/biolreprod.104.036277 15744026

[B11] Chamberlain A. J. Vander Jagt C. J. Hayes B. J. Khansefid M. Marett L. C. Millen C. A. (2015). Extensive Variation between Tissues in Allele Specific Expression in an Outbred Mammal. BMC Genomics 16, 993. 10.1186/s12864-015-2174-0 26596891 PMC4657355

[B12] Collier F. M. Gregorio-King C. C. Gough T. J. Talbot C. D. Walder K. Kirkland M. A. (2004). Identification and Characterization of a Lymphocytic Rho-GTPase Effector: Rhotekin-2. Biochem. Biophysical Res. Commun. 324, 1360–1369. 10.1016/j.bbrc.2004.09.205 15504364

[B13] Davuluri R. V. Suzuki Y. Sugano S. Zhang M. Q. (2000). CART Classification of Human 5' UTR Sequences. Genome Res. 10, 1807–1816. 10.1101/gr.gr-1460r 11076865 PMC310970

[B14] Dias M. M. Cánovas A. Mantilla-Rojas C. Riley D. G. Luna-Nevarez P. Coleman S. J. (2017). SNP Detection Using RNA-Sequences of Candidate Genes Associated with Puberty in Cattle. Genet. Mol. Res. 16. 10.4238/gmr16019522 28340271

[B16] Dobin A. Davis C. A. Schlesinger F. Drenkow J. Zaleski C. Jha S. (2013). STAR: Ultrafast Universal RNA-Seq Aligner. Bioinformatics 29, 15–21. 10.1093/bioinformatics/bts635 23104886 PMC3530905

[B17] Dongren R. Jun R. Yuyun X. Junwu M. Yanbo W. Yuanmei G. (2006). Mutatuion in the Porcine SERPINA7 Gene and its Association with Boar Fertility. Tpns 16, 1111–1114. 10.1080/10020070612330118

[B18] Dowsing A. T. Yong E. Clark M. Mclachlan R. I. De Kretser D. M. Trounson A. O. (1999). Linkage between Male Infertility and Trinucleotide Repeat Expansion in the Androgen-Receptor Gene. Lancet 354, 640–643. 10.1016/s0140-6736(98)08413-x 10466666

[B19] Feng S. Xu M. Liu F. Cui C. Zhou B. (2019). Reconstruction of the Full-Length Transcriptome Atlas Using PacBio Iso-Seq Provides Insight into the Alternative Splicing in Gossypium Australe. BMC Plant Biol. 19, 365. 10.1186/s12870-019-1968-7 31426739 PMC6701088

[B21] Fortes M. R. S. Lehnert S. A. Bolormaa S. Reich C. Fordyce G. Corbet N. J. (2012a). Finding Genes for Economically Important Traits: Brahman Cattle Puberty. Anim. Prod. Sci. 52, 143–150. 10.1071/an11165

[B23] Fortes M. R. S. Reverter A. Hawken R. J. Bolormaa S. Lehnert S. A. (2012b). Candidate Genes Associated with Testicular Development, Sperm Quality, and Hormone Levels of Inhibin, Luteinizing Hormone, and Insulin-like Growth Factor 1 in Brahman Bulls1. Biol. Reprod. 87 (3), 58. 10.1095/biolreprod.112.101089 22811567

[B22] Fortes M. R. S. Li Y. Collis E. Zhang Y. Hawken R. J. (2013a). The IGF1 Pathway Genes and Their Association with Age of Puberty in Cattle. Anim. Genet. 44, 91–95. 10.1111/j.1365-2052.2012.02367.x 22554198

[B20] Fortes M. R. S. Kemper K. Sasazaki S. Reverter A. Pryce J. E. Barendse W. (2013b). Evidence for Pleiotropism and Recent Selection in thePLAG1region in Australian Beef Cattle. Anim. Genet. 44, 636–647. 10.1111/age.12075 23909810

[B24] Forutan M. Ross E. Chamberlain A. J. Nguyen L. Mason B. Moore S. (2021). Evolution of Tissue and Developmental Specificity of Transcription Start Sites in Bos taurus indicus. Commun. Biol. 4, 829. 34211114 10.1038/s42003-021-02340-6 PMC8249380

[B26] Freiman R. N. Albright S. R. Zheng S. Sha W. C. Hammer R. E. Tjian R. (2001). Requirement of Tissue-Selective TBP-Associated Factor TAF II 105 in Ovarian Development. Science 293, 2084–2087. 10.1126/science.1061935 11557891

[B27] Fu Q. Yu L. Liu Q. Zhang J. Zhang H. Zhao S. (2000). Molecular Cloning, Expression Characterization, and Mapping of a Novel Putative Inhibitor of Rho GTPase Activity, RTKN, to D2S145-D2s286. Genomics 66, 328–332. 10.1006/geno.2000.6212 10873388

[B28] Gao Q. Wolfgang M. J. Neschen S. Morino K. Horvath T. L. Shulman G. I. (2004). Disruption of Neural Signal Transducer and Activator of Transcription 3 Causes Obesity, Diabetes, Infertility, and thermal Dysregulation. Proc. Natl. Acad. Sci. 101, 4661–4666. 10.1073/pnas.0303992101 15070774 PMC384803

[B29] Garrido-Martín D. Borsari B. Calvo M. Reverter F. Guigó R. (2021). Identification and Analysis of Splicing Quantitative Trait Loci across Multiple Tissues in the Human Genome. Nat. Commun. 12, 727. 10.1038/s41467-020-20578-2 33526779 PMC7851174

[B30] Gonzalez-Garay M. L. (2016). Introduction to Isoform Sequencing Using Pacific Biosciences Technology (Iso-Seq). Editor Wu J. (Dordrecht: Springer Netherlands), 141–160. 10.1007/978-94-017-7450-5_6:

[B31] Halder K. Wieland M. Hartig J. S. (2009). Predictable Suppression of Gene Expression by 5′-UTR-Based RNA Quadruplexes. Nucleic Acids Res. 37, 6811–6817. 10.1093/nar/gkp696 19740765 PMC2777418

[B32] Hayes B. J. Daetwyler H. D. (2019). 1000 Bull Genomes Project to Map Simple and Complex Genetic Traits in Cattle: Applications and Outcomes. Annu. Rev. Anim. Biosci. 7, 89–102. 10.1146/annurev-animal-020518-115024 30508490

[B33] Hecht N. Cavalcanti M. C. O. Nayudu P. Behr R. Reichenbach M. Weidner W. (2011). Protamine-1 Represents a Sperm Specific Gene Transcript: a Study in *Callithrix jacchus* and *Bos taurus* . Andrologia 43, 167–173. 10.1111/j.1439-0272.2009.01038.x 21486395

[B34] Jacob A. G. Smith C. W. J. (2017). Intron Retention as a Component of Regulated Gene Expression Programs. Hum. Genet. 136, 1043–1057. 10.1007/s00439-017-1791-x 28391524 PMC5602073

[B35] Juma A. R. Damdimopoulou P. E. Grommen S. V. H. Van De Ven W. J. M. De Groef B. (2016). Emerging Role of PLAG1 as a Regulator of Growth and Reproduction. J. Endocrinol. 228, R45–R56. 10.1530/joe-15-0449 26577933

[B36] Kaneko H. (2016). “Inhibin,” in Handbook of Hormones. Editors Takei Y. Ando H. Tsutsui K. (San Diego: Academic Press), 292–294. 10.1016/b978-0-12-801028-0.00187-2

[B37] Karim L. Takeda H. Lin L. Druet T. Arias J. A. C. Baurain D. (2011). Variants Modulating the Expression of a Chromosome Domain Encompassing PLAG1 Influence Bovine Stature. Nat. Genet. 43, 405–413. 10.1038/ng.814 21516082

[B38] Kent J. Wheatley S. C. Andrews J. E. Sinclair A. H. Koopman P. (1996). A Male-specific Role for SOX9 in Vertebrate Sex Determination. Development 122, 2813–2822. 10.1242/dev.122.9.2813 8787755

[B39] Kim S. J. Park K. Koeller D. Kim K. Y. Wakefield L. M. Sporn M. B. (1992). Post-transcriptional Regulation of the Human Transforming Growth Factor-Beta 1 Gene. J. Biol. Chem. 267, 13702–13707. 10.1016/s0021-9258(18)42270-3 1618868

[B41] Lamorte W. W. (2016). Hypothesis Testing - Chi Squared Test [Online]. Available at: https://sphweb.bumc.bu.edu/otlt/MPH-Modules/BS/BS704_HypothesisTesting-ChiSquare/BS704_HypothesisTesting-ChiSquare3.html .

[B42] Lawless C. Pearson R. D. Selley J. N. Smirnova J. B. Grant C. M. Ashe M. P. (2009). Upstream Sequence Elements Direct post-transcriptional Regulation of Gene Expression Under Stress Conditions in Yeast. BMC Genom. 10, 7. 10.1186/1471-2164-10-7 PMC2649001 19128476

[B43] Le Reith D. (1997). Seminars in Medicine of the Beth Israel Deaconess Medical Center. Insulin-like Growth Factors. N. Engl. J. Med. 336, 633–640. 10.1056/NEJM199702273360907 9032050

[B44] Leppek K. Das R. Barna M. (2018). Functional 5′ UTR mRNA Structures in Eukaryotic Translation Regulation and How to Find Them. Nat. Rev. Mol. Cell Biol. 19, 158–174. 10.1038/nrm.2017.103 29165424 PMC5820134

[B45] Li H. Durbin R. (2009). Fast and Accurate Short Read Alignment with Burrows-Wheeler Transform. Bioinformatics 25, 1754–1760. 10.1093/bioinformatics/btp324 19451168 PMC2705234

[B46] Li H. Handsaker B. Wysoker A. Fennell T. Ruan J. Homer N. (2009). The Sequence Alignment/Map Format and SAMtools. Bioinformatics 25, 2078–2079. 10.1093/bioinformatics/btp352 19505943 PMC2723002

[B47] Li H. (2018). Minimap2: Pairwise Alignment for Nucleotide Sequences. Bioinformatics 34, 3094–3100. 10.1093/bioinformatics/bty191 29750242 PMC6137996

[B48] Littlejohn M. Grala T. Sanders K. Walker C. Waghorn G. Macdonald K. (2012). Genetic Variation in PLAG1 Associates with Early Life Body Weight and Peripubertal Weight and Growth in *Bos taurus* . Anim. Genet. 43, 591–594. 10.1111/j.1365-2052.2011.02293.x 22497486

[B49] Lyons R. E. Loan N. T. Dierens L. Fortes M. R. S. Kelly M. Mcwilliam S. S. (2014). Evidence for Positive Selection of Taurine Genes within a QTL Region on Chromosome X Associated with Testicular Size in Australian Brahman Cattle. BMC Genet. 15, 6. 10.1186/1471-2156-15-6 24410912 PMC3893399

[B50] Maclean H. E. Warne G. L. Zajac J. D. (1995). Defects of Androgen Receptor Function: from Sex Reversal to Motor Neurone Disease. Mol. Cell Endocrinol. 112, 133–141. 10.1016/0303-7207(95)03608-a 7489816

[B51] Malven P. (1995). Role of Endogenous Opioids for Regulation of the Oestrous Cycle in Sheep and Cattle. Reprod. Domest. Anim. 30, 183–187. 10.1111/j.1439-0531.1995.tb00143.x

[B52] Marques P. Skorupskaite K. Rozario K. S. Anderson R. A. George J. T. (2000). “Physiology of GnRH and Gonadotropin Secretion,” in Endotext. Editors Feingold K. R. Anawalt B. Boyce A. Chrousos G. de Herder W. W. Dhatariya K. (South Dartmouth (MA): MDText.com, Inc). Available at: https://www.ncbi.nlm.nih.gov/books/NBK279070/ .

[B53] Mcgowan K. A. Li J. Z. Park C. Y. Beaudry V. Tabor H. K. Sabnis A. J. (2008). Ribosomal Mutations Cause P53-Mediated Dark Skin and Pleiotropic Effects. Nat. Genet. 40, 963–970. 10.1038/ng.188 18641651 PMC3979291

[B55] Metcalf C. E. Wassarman D. A. (2007). Nucleolar Colocalization of TAF1 and Testis-specific TAFs duringDrosophilaspermatogenesis. Dev. Dyn. 236, 2836–2843. 10.1002/dvdy.21294 17823958

[B56] Minten M. A. Bilby T. R. Bruno R. G. S. Allen C. C. Madsen C. A. Wang Z. (2013). Effects of Fertility on Gene Expression and Function of the Bovine Endometrium. PLoS One 8, e69444. 10.1371/journal.pone.0069444 23940519 PMC3734181

[B57] Momboisse F. Houy S. Ory S. Calco V. Bader M. F. Gasman S. (2011). How Important Are Rho GTPases in Neurosecretion? J. Neurochem. 117, 623–631. 10.1111/j.1471-4159.2011.07241.x 21392006

[B58] Moore S. G. Pryce J. E. Hayes B. J. Chamberlain A. J. Kemper K. E. Berry D. P. (2016). Differentially Expressed Genes in Endometrium and Corpus Luteum of Holstein Cows Selected for High and Low Fertility Are Enriched for Sequence Variants Associated with Fertility1. Biol. Reprod. 94. 10.1095/biolreprod.115.132951 26607721

[B59] Mota R. R. Guimarães S. E. F. Fortes M. R. S. Hayes B. Silva F. F. Verardo L. L. (2017). Genome-wide Association Study and Annotating Candidate Gene Networks Affecting Age at First Calving in Nellore Cattle. J. Anim. Breed. Genet. 134, 484–492. 10.1111/jbg.12299 28994157

[B60] Müller M.-P. Rothammer S. Seichter D. Russ I. Hinrichs D. Tetens J. (2017). Genome-wide Mapping of 10 Calving and Fertility Traits in Holstein Dairy Cattle with Special Regard to Chromosome 18. J. Dairy Sci. 100, 1987–2006. 10.3168/jds.2016-11506 28109604

[B61] National Center for Biotechnology Information (2008). BLAST® Command Line Applications User Manual [Online].

[B62] Nguyen L. Reverter A. Cánovas A. Porto-Neto L. Venus B. Islas-Trejo A. (2019). Pre-and Post-Puberty Co-expression Gene Networks in Brahman Heifers Using RNA-Sequencing.

[B63] Nguyen L. T. Reverter A. Cánovas A. Venus B. Anderson S. T. Islas-Trejo A. (2018). STAT6, PBX2, and PBRM1 Emerge as Predicted Regulators of 452 Differentially Expressed Genes Associated with Puberty in Brahman Heifers. Front. Genet. 9. 10.3389/fgene.2018.00087 PMC5869259 29616079

[B64] Nguyen L. T. Reverter A. Cánovas A. Venus B. Islas-Trejo A. Porto-Neto L. R. (2017). Global Differential Gene Expression in the Pituitary Gland and the Ovaries of Pre- and Postpubertal Brahman Heifers1. J. Anim. Sci. 95, 599–615. 10.2527/jas.2016.0921 28380590

[B65] Nonneman D. Rohrer G. A. Wise T. H. Lunstra D. D. Ford J. J. (2005). A Variant of Porcine Thyroxine-Binding Globulin Has Reduced Affinity for Thyroxine and Is Associated with Testis Size1. Biol. Reprod. 72, 214–220. 10.1095/biolreprod.104.031922 15385420

[B66] O'leary N. A. Wright M. W. Brister J. R. Ciufo S. Haddad D. Mcveigh R. (2016). Reference Sequence (RefSeq) Database at NCBI: Current Status, Taxonomic Expansion, and Functional Annotation. Nucleic Acids Res. 44, D733–D745. 10.1093/nar/gkv1189 26553804 PMC4702849

[B67] Ozsolak F. Milos P. M. (2011). RNA Sequencing: Advances, Challenges and Opportunities. Nat. Rev. Genet. 12, 87–98. 10.1038/nrg2934 21191423 PMC3031867

[B68] Pausch H. Flisikowski K. Jung S. Emmerling R. Edel C. Götz K.-U. (2011). Genome-wide Association Study Identifies Two Major Loci Affecting Calving Ease and Growth-Related Traits in Cattle. Genetics 187, 289–297. 10.1534/genetics.110.124057 21059885 PMC3018322

[B69] Pesole G. Mignone F. Gissi C. Grillo G. Licciulli F. Liuni S. (2001). Structural and Functional Features of Eukaryotic mRNA Untranslated Regions. Gene 276, 73–81. 10.1016/s0378-1119(01)00674-6 11591473

[B70] Pleiss J. A. Whitworth G. B. Bergkessel M. Guthrie C. (2007). Rapid, Transcript-specific Changes in Splicing in Response to Environmental Stress. Mol. Cell 27, 928–937. 10.1016/j.molcel.2007.07.018 17889666 PMC2081968

[B71] Poppe K. Velkeniers B. Glinoer D. (2008). The Role of Thyroid Autoimmunity in Fertility and Pregnancy. Nat. Rev. Endocrinol. 4, 394–405. 10.1038/ncpendmet0846 18506157

[B72] Richards J. S. Pangas S. A. (2010). The Ovary: Basic Biology and Clinical Implications. J. Clin. Invest. 120, 963–972. 10.1172/jci41350 20364094 PMC2846061

[B73] Robinson A. J. Ross E. M. (2019). QuAdTrim: Overcoming Computational Bottlenecks in Sequence Quality Control. bioRxiv. 10.1101/2019.12.18.870642

[B74] Robinson J. T. Thorvaldsdóttir H. Winckler W. Guttman M. Lander E. S. Getz G. (2011). Integrative Genomics Viewer. Nat. Biotechnol. 29, 24–26. 10.1038/nbt.1754 21221095 PMC3346182

[B75] Rosen B. D. Bickhart D. M. Schnabel R. D. Koren S. Elsik C. G. Tseng E. (2020). De Novo assembly of the Cattle Reference Genome with Single-Molecule Sequencing. GigaScience 9, giaa021. 10.1093/gigascience/giaa021 32191811 PMC7081964

[B76] Ross E. M. Nguyen L. T. Lamb H. J. Moore S. S. Hayes B. J. (2022). The Genome of Tropically Adapted Brahman Cattle (*Bos taurus indicus*) Reveals Novel Genome Variation in Production Animals. bioRxiv. 10.1101/2022.02.09.479458

[B77] Salavati M. Caulton A. Clark R. Gazova I. Smith T. P. L. Worley K. C. (2020). Global Analysis of Transcription Start Sites in the New Ovine Reference Genome (Oar Rambouillet v1.0). Front. Genet. 11. 10.3389/fgene.2020.580580 PMC7645153 33193703

[B78] Simmen R. C. M. Ko Y. Simmen F. A. (1993). Insulin-like Growth Factors and Blastocyst Development. Theriogenology 39, 163–175. 10.1016/0093-691x(93)90031-y

[B79] Snelling W. M. Allan M. F. Keele J. W. Kuehn L. A. Mcdaneld T. Smith T. P. L. (2010). Genome-wide Association Study of Growth in Crossbred Beef Cattle12. J. Anim. Sci. 88, 837–848. 10.2527/jas.2009-2257 19966163

[B80] Soares A. C. C. Guimarães S. E. F. Kelly M. J. Fortes M. R. S. e Silva F. F. Verardo L. L. (2017). Multiple-trait Genomewide Mapping and Gene Network Analysis for Scrotal Circumference Growth Curves in Brahman Cattle. J. Anim. Sci. 95, 3331–3345. 10.2527/jas2017.1409 28805926

[B81] Steilmann C. Paradowska A. Bartkuhn M. Vieweg M. Schuppe H.-C. Bergmann M. (2011). Presence of Histone H3 Acetylated at Lysine 9 in Male Germ Cells and its Distribution Pattern in the Genome of Human Spermatozoa. Reprod. Fertil. Dev. 23, 997–1011. 10.1071/rd10197 22127005

[B82] Stelzer G. Rosen N. Plaschkes I. Zimmerman S. Twik M. Fishilevich S. (2016). The GeneCards Suite: From Gene Data Mining to Disease Genome Sequence Analyses. Curr. Protoc. Bioinformatics 54, 1–33.30.31-31.30.33. 10.1002/cpbi.5 27322403

[B83] Syed N. H. Kalyna M. Marquez Y. Barta A. Brown J. W. S. (2012). Alternative Splicing in Plants - Coming of Age. Trends Plant Sci. 17, 616–623. 10.1016/j.tplants.2012.06.001 22743067 PMC3466422

[B84] Takahashi A. (2016). “Enkephalin,” in Handbook of Hormones. Editors Takei Y. Ando H. Tsutsui K. (San Diego, CA: Academic Press), 55–e7AA-52. 10.1016/b978-0-12-801028-0.00117-3 :

[B85] Takahashi H. Kato S. Murata M. Carninci P. (2012a). CAGE (Cap Analysis of Gene Expression): a Protocol for the Detection of Promoter and Transcriptional Networks. Methods Mol. Biol. 786, 181–200. 10.1007/978-1-61779-292-2_11 21938627 PMC4094367

[B86] Takahashi H. Lassmann T. Murata M. Carninci P. (2012b). 5′ End-Centered Expression Profiling Using Cap-Analysis Gene Expression and Next-Generation Sequencing. Nat. Protoc. 7, 542–561. 10.1038/nprot.2012.005 22362160 PMC4094379

[B87] Taylor J. A. Goubillon M.-L. Broad K. D. Robinson J. E. (2007). Steroid Control of Gonadotropin-Releasing Hormone Secretion: Associated Changes in Pro-opiomelanocortin and Preproenkephalin Messenger RNA Expression in the Ovine Hypothalamus1. Biol. Reprod. 76, 524–531. 10.1095/biolreprod.106.055533 17151352

[B88] Thomsen M. K. Francis J. C. Swain A. (2008). The Role of Sox9 in Prostate Development. Differentiation 76, 728–735. 10.1111/j.1432-0436.2008.00293.x 18557758

[B89] Ulloa-Aguirre A. Reiter E. Crépieux P. (2018). FSH Receptor Signaling: Complexity of Interactions and Signal Diversity. Endocrinology 159, 3020–3035. 10.1210/en.2018-00452 29982321

[B91] Utsunomiya Y. T. Milanesi M. Utsunomiya A. T. H. Torrecilha R. B. P. Kim E.-S. Costa M. S. (2017). A PLAG1 Mutation Contributed to Stature Recovery in Modern Cattle. Sci. Rep. 7, 17140. 10.1038/s41598-017-17127-1 29215042 PMC5719367

[B92] Van Der Velden A. W. Thomas A. A. M. (1999). The Role of the 5′ Untranslated Region of an mRNA in Translation Regulation during Development. Int. J. Biochem. Cell Biol. 31, 87–106. 10.1016/s1357-2725(98)00134-4 10216946

[B93] Velazquez M. A. Spicer L. J. Wathes D. C. (2008). The Role of Endocrine Insulin-like Growth Factor-I (IGF-I) in Female Bovine Reproduction. Domest. Anim. Endocrinol. 35, 325–342. 10.1016/j.domaniend.2008.07.002 18703307

[B94] Vidal V. P. I. Chaboissier M.-C. De Rooij D. G. Schedl A. (2001). Sox9 Induces Testis Development in XX Transgenic Mice. Nat. Genet. 28, 216–217. 10.1038/90046 11431689

[B95] Wagner M. S. Wajner S. M. Maia A. L. (2008). The Role of Thyroid Hormone in Testicular Development and Function. J. Endocrinol. 199, 351–365. 10.1677/joe-08-0218 18728126 PMC2799043

[B96] Wang R.-S. Yeh S. Tzeng C.-R. Chang C. (2009). Androgen Receptor Roles in Spermatogenesis and Fertility: Lessons from Testicular Cell-specific Androgen Receptor Knockout Mice. Endocr. Rev. 30, 119–132. 10.1210/er.2008-0025 19176467 PMC2662628

[B97] Wang Z. Gerstein M. Snyder M. (2009). RNA-seq: a Revolutionary Tool for Transcriptomics. Nat. Rev. Genet. 10, 57–63. 10.1038/nrg2484 19015660 PMC2949280

[B98] Wilson M. (1998). Premature Elevation in Serum Insulin-like Growth Factor-I Advances First Ovulation in Rhesus Monkeys. J. Endocrinol. 158, 247–257. 10.1677/joe.0.1580247 9771469

[B99] Xie S. Q. Han Y. Chen X. Z. Cao T. Y. Ji K. K. Zhu J. (2018). ISOdb: A Comprehensive Database of Full-Length Isoforms Generated by Iso-Seq. Int. J. Genomics 2018, 9207637. 10.1155/2018/9207637 30581839 PMC6276398

[B100] Yeh S. Tsai M.-Y. Xu Q. Mu X.-M. Lardy H. Huang K.-E. (2002). Generation and Characterization of Androgen Receptor Knockout (ARKO) Mice: An *In Vivo* Model for the Study of Androgen Functions in Selective Tissues. Proc. Natl. Acad. Sci. 99, 13498–13503. 10.1073/pnas.212474399 12370412 PMC129702

[B101] Yilmaz A. Davis M. E. Simmen R. C. M. (2006). Analysis of Female Reproductive Traits in Angus Beef Cattle Divergently Selected for Blood Serum Insulin-Like Growth Factor I Concentration. Theriogenology 65, 1180–1190. 10.1016/j.theriogenology.2005.06.018 16144706

[B102] Yilmaz A. Davis M. E. Simmen R. C. M. (2004). Estimation of (Co)variance Components for Reproductive Traits in Angus Beef Cattle Divergently Selected for Blood Serum IGF-I Concentration123. J. Anim. Sci. 82, 2285–2292. 10.2527/2004.8282285x 15318726

[B103] Yu P. Ma D. Xu M. (2005). Nested Genes in the Human Genome. Genomics 86, 414–422. 10.1016/j.ygeno.2005.06.008 16084061

[B105] Zhang Z. Schwartz S. Wagner L. Miller W. (2000). A Greedy Algorithm for Aligning DNA Sequences. J. Comput. Biol. 7, 203–214. 10.1089/10665270050081478 10890397

