# Supplementary material for: Extensive Variation in Gene Expression is Revealed in 13 Fertility-Related Genes Using RNA-Seq, ISO-Seq, and CAGE-Seq From Brahman Cattle
Source: Front Genet. 2022 Mar 25;13:784663. doi: 10.3389/fgene.2022.784663 (PMC8990236; doi:10.3389/fgene.2022.784663)
Supplement: Supplementary file 1 [file DataSheet4.docx]

| Gene | Isoforms | Bos taurus | | | Bos indicus | | | Length of gene ^a^ | Chromosome | Strand direction ^b^ | | Homology (%) | | |
| --- | --- | --- | --- | --- | --- | --- | --- | --- | --- | --- | --- | --- | --- | --- |
|  |  | Start Position | Stop Position | Start Position | | Stop Position |  | |  | |  | |  |  |
| AR | X1 | 51674157 | 51881942 | 84786064 | | 84957236 | 171172 | | X | | Negative | | 99.96 |  |
| IGF | X1 | 66206081 | 66263849 | 66145894 | | 66203733 | 57839 | | 5 | | Negative | | 100 |  |
|  | X2 | 66206081 | 66263849 | 66145894 | | 66203733 | 57839 | |  | |  | | 99.65 |  |
|  | X3 | 66206081 | 66261980 | 66145894 | | 66199198 | 53304 | |  | |  | | 100 |  |
|  | X4 | 66206081 | 66261980 | 66145894 | | 66199195 | 53301 | |  | |  | | 100 |  |
|  | X5 | 66192424 | 66263849 | 66132237 | | 66203733 | 71496 | |  | |  | | 99.37 |  |
|  | X6 | 66192595 | 66263849 | 66132408 | | 66147706 | 15298 | |  | |  | | 99.33 |  |
|  | X7 | 66192424 | 66261980 | 66132237 | | 66147706 | 15469 | |  | |  | | 99.76 |  |
|  | X8 | 66192424 | 66261980 | 66132237 | | 66147706 | 15469 | |  | |  | | 99.75 |  |
|  | Preprotein | 66192424 | 66263849 | 66132237 | | 66147706 | 15469 | |  | |  | | 99.79 |  |
| INHA | X1 | 107501844 | 107504762 | 107614722 | | 107617642 | 2920 | | 2 | | Positive | | 99.82 |  |
| PENK | X1 | 23542677 | 23546157 | 23420291 | | 23423774 | 3483 | | 14 | | Negative | | 100 |  |
| PLAG1 | X1 | 23330541 | 23332546 | 23192090 | | 23194095 | 2005 | | 14 | | Negative | | 100 |  |
|  | X2 | 23330541 | 23331794 | 23192090 | | 23193343 | 1253 | |  | |  | |  |  |
| RTKN2 | X1 | 18284769 | 18393694 | 17858619 | | 17967072 | 108453 | | 28 | | Negative | | 99.3 |  |
|  | X2 | 18284769 | 18385527 | 17858619 | | 17958855 | 100236 | |  | |  | | 99.26 |  |
|  | X3 | 18284769 | 18348138 | 17858619 | | 17921964 | 63345 | |  | |  | | 99.33 |  |
| SERPINA7 | X1 | 54824445 | 54829800 | 53237946 | | 53241468 | 3522 | | X | | Positive | | 99.69 |  |
|  | Precursor | 54824445 | 54827949 | 53237963 | | 53241468 | 3505 | |  | |  | | 99.68 |  |
| SOX9 | X1 | 58919579 | 58922699 | 60166722 | | 60169843 | 3121 | | 19 | | Negative | | 100 |  |
| STAT3 | X1 | 42419849 | 42450618 | 43645371 | | 43676141 | 30770 | | 19 | | Negative | | 99.66 |  |
|  | X2 | 42419849 | 42450618 | 43645371 | | 43676141 | 30770 | |  | |  | | 99.66 |  |
|  | X3 | 42421282 | 42450618 | 43647176 | | 43676141 | 28965 | |  | |  | | 99.64 |  |
| STK11IP | X1 | 107521189 | 107535476 | 107634090 | | 107648385 | 14295 | | 2 | | Positive | | 99.45 |  |
|  | X2 | 107521189 | 107535476 | 107634090 | | 107648385 | 11551 | |  | |  | | 99.5 |  |
|  | X3 | 107521189 | 107533477 | 107634090 | | 107646987 | 12297 | |  | |  | | 99.46 |  |
| TAF1 | X1 | 79206804 | 79276760 | 80853773 | | 80923837 | 70064 | | X | | Negative | | 99.86 |  |
|  | X2 | 79206804 | 79276760 | 80853773 | | 80923837 | 70064 | |  | |  | | 99.86 |  |
|  | X3 | 79206804 | 79276760 | 80853773 | | 80923837 | 70064 | |  | |  | | 99.79 |  |
|  | X4 | 79206804 | 79276760 | 80853773 | | 80923837 | 70064 | |  | |  | | 99.84 |  |
|  | X5 | 79206804 | 79276760 | 80853773 | | 80923837 | 70064 | |  | |  | | 99.84 |  |
|  | X6 | 79197983 | 79276760 | 80857229 | | 80923837 | 66608 | |  | |  | | 99.87 |  |
|  | X7 | 79225602 | 79276760 | 80872738 | | 80923837 | 51099 | |  | |  | | 99.9 |  |
|  | X8 | 79229398 | 79276760 | 80885675 | | 80923837 | 38162 | |  | |  | | 99.9 |  |
|  | X9 | 79227390 | 79276760 | 80885675 | | 80923837 | 38162 | |  | |  | | 99.9 |  |
| TAF9B | X1 | 74232003 | 74255254 | 74070058 | | 74093312 | 23254 | | X | | Positive | | 100 |  |
|  | X2 | 74232003 | 74255924 | 74070058 | | 74093982 | 23924 | |  | |  | | 100 |  |
|  | Subunit | 74232003 | 74239263 | 74070058 | | 74077335 | 7277 | |  | |  | | 100 |  |
