# Supplementary material for: Extensive Variation in Gene Expression is Revealed in 13 Fertility-Related Genes Using RNA-Seq, ISO-Seq, and CAGE-Seq From Brahman Cattle
Source: Front Genet. 2022 Mar 25;13:784663. doi: 10.3389/fgene.2022.784663 (PMC8990236; doi:10.3389/fgene.2022.784663)

Supplementary material

Supplementary Table S1. Summary of raw reads generated for each library from IsoSeq, RNAseq and CAGEseq sequencing

	**Isoseq**			**RNA seq**	**CAGEseq**
	ZMW*	Full-length reads	Mapped reads	Raw reads	Raw reads
**Fetal Liver**	557,868	205,952	205,904	78,492,675	15,141,599
**Fetal Lung**	519,277	394,189	394,170	90,713,379	2,944,730
**Liver**	459,570	342,585	342,517	77,648,618	19,113,216
**Lung**	NA**	NA**	NA**	69,662,155	37,473,013
**Blood**	574,977	410,796	408,585	68,392,292	4,986,447
**Thyroid**	570,816	403,006	402,991	67,905,471	18,954,871
**Spleen**	486,355	376,649	376,635	74,322,017	6,164,132
**Kidney**	521,514	370,574	370,550	76,661,390	20,244,459
**Muscle**	315,986	211,985	211,953	80,903,660	7,414,926
**Ovary**	490,676	364,170	364,116	NA**	12,315,069
**Uterus**	530,312	394,387	394,307	NA**	12,211,715

* ZMW means Zero mode waveguide

** NA means sequencing data is not available

Supplementary Table S2. Summary of read length for each tissue generated from ISOseq sequencing

Library	Average read length	Maximum length
Blood	1647	6696
Foetal Lung	1847	6997
Foetal Liver	1563	6178
Kidney	2056	10305
Liver	1438	6284
Muscle	1375	5508
Ovary	1381	4557
Spleen	1949	8202
Thyroid	2141	7474
Uterus	1619	10096

INTRON SPANNING READS FROM RNA-SEQ DATA

RNAseq intron spanning reads were used to support the presence of novel isoform. RNAseq alignments were visualised in IGV and intron spanning read counts were extracted directly from the alignments.

Supplementary Table S3. Intron spanning read for IGF1 from RNAseq data.

	INTRON 1	INTRON 2	INTRON 3
**Spleen**	87	90	82
**Thyroid**	70	74	77

Supplementary Table S4. Intron spanning read for RPS20 from RNAseq data.

	INTRON 1	INTRON 2	INTRON 3
**Kidney**	1062	4304	4295
**Blood**	3190	13110	12963
**Foetal liver**	2737	11499	11892
**Foetal lung**	1961	7668	7864
**Liver**	844	3128	3386
**Muscle**	1231	5070	5054
**Spleen**	4954	19253	19210
**Thyroid**	6169	23903	24789

Supplementary Table S5. Intron spanning read for RTKN2 from RNAseq data.

	1	2	3	4	5	6	7	8	9	10	11
**Foetal lung**	1	20	12	26	19	10	5	14	22	21	11
**Thyroid**	6	10	5	12	7	1	5	4	4	5	4
**Spleen**	-	-	2	2	2	4	1	2	3	2	1

Supplementary Table S6. Intron spanning read for SERPINA7 from RNAseq data.

	INTRON 1	INTRON 2	INTRON 3	INTRON 4
**Foetal liver**	1505	1297	1526	321
**Foetal lung**	533	395	446	87

Supplementary Figure S1: Histogram shows the size distribution of all ISO-seq libraries.

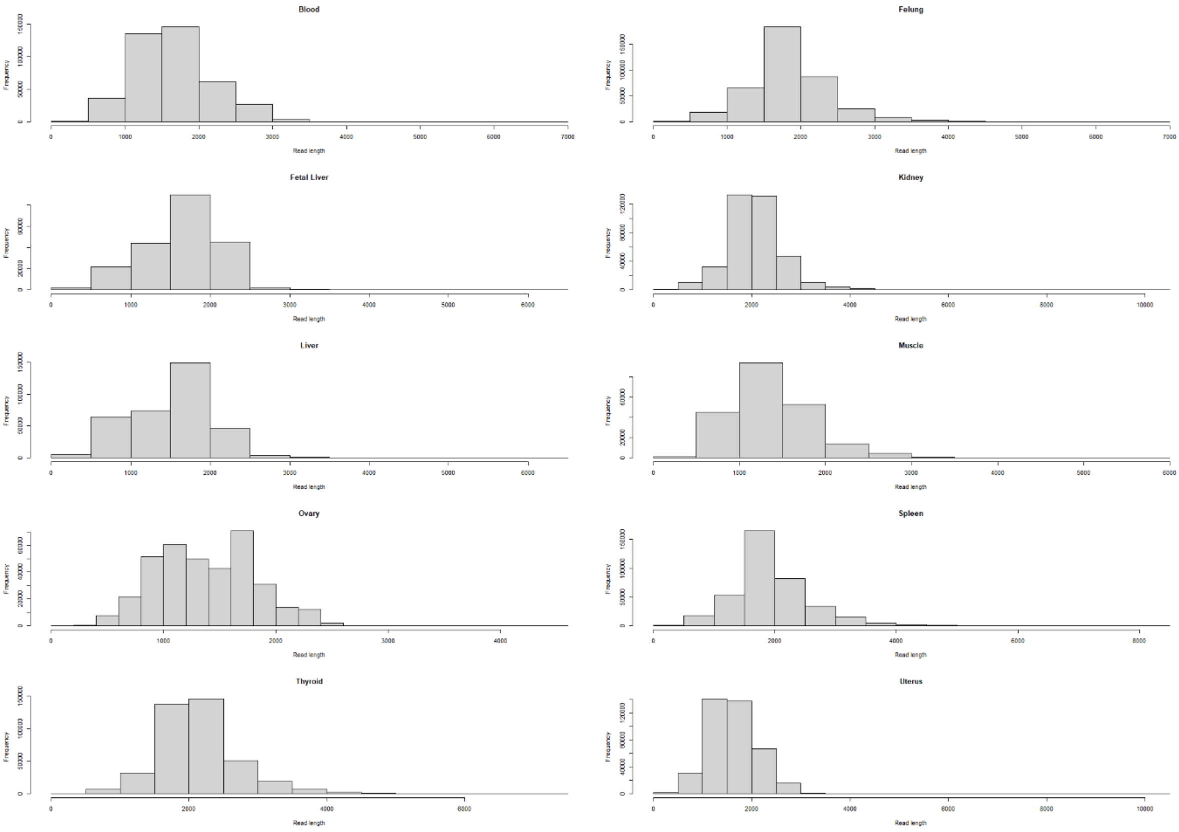

Supplement: Supplementary file 3 [file DataSheet3.pdf]
